# Use of Injectable Platelet-Rich Fibrin Accompanied by Bone Graft in Socket Endurance: A Radiographic and Histological Study

**DOI:** 10.7759/cureus.46909

**Published:** 2023-10-12

**Authors:** Tanya Nagrani, Santosh Kumar, Md. Ahsanul Haq, Sivaraman Dhanasekaran, Shreya Gajjar, Chandni Patel, Susmita Sinha, Mainul Haque

**Affiliations:** 1 Periodontology, Karnavati School of Dentistry, Karnavati University, Gandhinagar, IND; 2 Periodontology and Implantology, Karnavati School of Dentistry, Karnavati University, Gandhinagar, IND; 3 Bio-Statistics, Infectious Diseases Division, icddr, b, Dhaka, BGD; 4 School of Energy Technology, Pandit Deendayal Energy University, Gandhinagar, IND; 5 Physiology, Khulna City Medical College and Hospital, Khulna, BGD; 6 Karnavati Scientific Research Center (KSRC), Karnavati School of Dentistry, Karnavati University, Gandhinagar, IND; 7 Pharmacology and Therapeutics, National Defence University of Malaysia, Kuala Lumpur, MYS

**Keywords:** demineralized freeze-dried bone allografts, platelet-rich-fibrin, fdba, freeze-dried bone allograft, i-prf, injectable prf, bone allograft, socket preservation, socket plug technique, bone morphogenic protein

## Abstract

Background

Ridge preservation became a crucial dental health issue and strategy to keep away from ridge defacement after post-tooth loss. The recent scientific evolution of platelet-rich fibrin (PRF) comprises a parenteral formulation of PRF. The combined allograft for socket preservation gives benefits. In this study, bone allografts, demineralized freeze-dried bone allografts (DFDBA) and freeze-dried bone allografts (FDBA) are used in a 30:70 ratio alone or in combination with injectable PRF (I-PRF) for socket preservation.

Methods

This study is a radiographic and histological examination conducted on 60 participants aged between 19-65 years. Participating patients agreed voluntarily that they would not bear any fixed prosthesis for the next nine months and plan for implanted teeth placement, including multi-rooted mandibular molars denticles. Both groups received atraumatic extraction; then, the socket was preserved with bone allograft alone in the control group and bone allograft mixed with I-PRF, forming sticky bone, in the experimental group. Clinical, radiological, and histological assessments were taken at the inception stage, three months, six months, and nine months. A multivariate regression model and a generalized estimating equation (GEE) model were used to analyse the effects of these changes on outcomes.

Results

In all the parameters, the test group indicated a good amount of bone growth with increasing intervals of time for bone height radiographically with statistically significant difference present (p<0.05) and histologically after nine months when socket site grafted with bone graft in combination with I-PRF.

Conclusion

This study's results demonstrated that I-PRF possesses the potential to regenerate and heal in the tooth-extracted socket. This study further recommends the implementation of I-PRF in safeguarding and conserving the raised rim of the tooth. Future research should take place on the osteogenic capability of I-PRF in more comprehensive ridge accession surgical procedures and additional expanding and improving capacities in periodontal reconstruction.

## Introduction

It has been reported that most dental extractions are done without respect for the alveolar ridge preservation [1-4]. They are thus one of the most common causes of dimensions and morphological changes in the alveolar ridge [5-7]. Many experimental studies have demonstrated that the coronal section of the dental extraction sockets of the buccal bone wall commonly lacks bundle bone [8,9]. After that, tooth extraction often leads to poor functional ability of the bundle bone [6,10,11]. It is resorbed due to osteoclastic activity, which changes the buccal crest dimensions. The tooth socket's buccal wall is usually resorbed partially or vertically and horizontally, resulting in bucco-oral alterations [12,13].

Multiple researchers have coined the "socket plug" technique to describe various socket protection techniques [14-18]. The method is divided into atraumatic tooth extractions, a conservative flap design, biomaterial implantation, and suturing [14]. There was a notable presence of osteoclasts in the section of the alveolar ridge that is unveiled [19,20], showing surface resorption symptoms in the initial few weeks of healing dental patients [15,21]. This could also elucidate the dimensional shifts following tooth extraction [22-24]. As a result, preserving the tooth extraction socket becomes a critical responsibility that can be accomplished through various methods [25,26]; however, it must also ensure long-term bone volume stability and be based on reliable research [7,27].

There is a higher reduction of the highest point of the dental crest in the mandibular than in the maxillary [28-30]. In contrast, ridge width loss is considerably more on the buccal plate in the mandibular and maxillary situates [28,30]. Post-extraction resorption is associated with thinner buccal plates [11,31,32]. Erstwhile researchers have found that a complete mucoperiosteal flap causes crestal bone loss [33-36], which could be linked to bone loss after tooth extraction [37,38].

Platelet-rich fibrin (PRF) has grown in popularity through the last decennium and is now used in several dentistry and medical procedures [39-41]. PRF is used in dentistry for extraction socket preservation, gingival recession treatment, intrabony defects, periodontal defect regeneration, and hyperplastic gingival tissues [42,43].

The latest scientific progress in PRF is injectable PRF (I-PRF) [44,45]. Previously conducted research reported that possessing shortcomings of PRF over platelet-rich plasma (PRP) is that it is obtained only in a gel form, which limits its applications [46,47]. Consequently, it is not suitable for that clinical condition requiring injections. A liquid form of I-PRF has recently been created and remains liquid for 15-20 minutes [48,49]. I-PRF currently serves as one kind of human tissue regeneration [40]. I-PRF has progressed by injecting corresponding homologized PRF into patients' damaged soft tissue, mucous membranes, or skin [48,50].

Some researchers have combined I-PRF with bone graft particles to improve the biotic and material features of the implanted substances [51,52]. The amalgamation or blended version has proven advantages, including increased angiogenesis and outstanding therapeutic capabilities by merging the minute bone grains into an immense substance for bone grafting operative procedures [53-56]. Thus, additional bone grafts with I-PRF have a considerable therapeutic effect in enhancing the dental socket repair process [56,57]. Most preferred bone grafts are allograft, xenograft, and alloplast [58,59].

Various types of bone allografts such as freeze-dried bone allografts (FDBA), demineralized freeze-dried bone allografts (DFDBA), and fresh-frozen bone allografts (FFBA) are available [60,61]. The use of FFBA and DFDBA has been minimized because of immune response complications, which were previously well-known in relation to fresh-frozen bone [62-64]. Nowadays, FFBA and DFDBA are the most often utilized allografts for dental crest safeguarding [30,65-67]. FDBA revascularization ensues at the acquirable patient site by coalescing or restoring (creeping swapping) and evolving connective tissue regions [65]. DFDBA primarily comprises collagen but contains other proteins, such as bone morphogenetic proteins (BMPs) and osteoinductive bone components [68,69]. Additional growth factors are present, such as platelet-derived and transforming growth factors [70,71]. BMP has been proven to increase the differentiation of osteoblasts [72,73]. A combination of different allografts for dental extraction cavity preserved better than a single graft and benefited both the patient and treatment strategy, as blended graft results in volume stability and improved bone quality and composition [74]. A study revealed that coalescence allografts show better clinical outcomes with regard to maintaining ridge features (both height and width) and graft unification [75].

This study combined different grafts and compared their impact with I-PRF on alveolar ridge preservation. Dental ridge preservation is critical because, after tooth extraction, the socket forms, causing ridge destruction. This bone loss further promotes difficulty in mastication, speaking, socialization, and lifestyle disorders [1,65,76,77]. Furthermore, the current study is designed to appraise and assess the equivalence of the histological, radiographic, and clinical parameters of using I-PRF with bone or bone graft to preserve extraction sockets. The objectives were to assess the preservation of the socket site next to extraction, protection following single-tooth extraction site using combination graft (DFDBA and FDBA), preservation following single-tooth extraction using I-PRF gel and combination graft (DFDBA+FDBA), analyzing the use of bone graft alone or in a mixture of bone graft and I-PRF.

## Materials and methods

Study design and patient criteria

**Figure 1:**
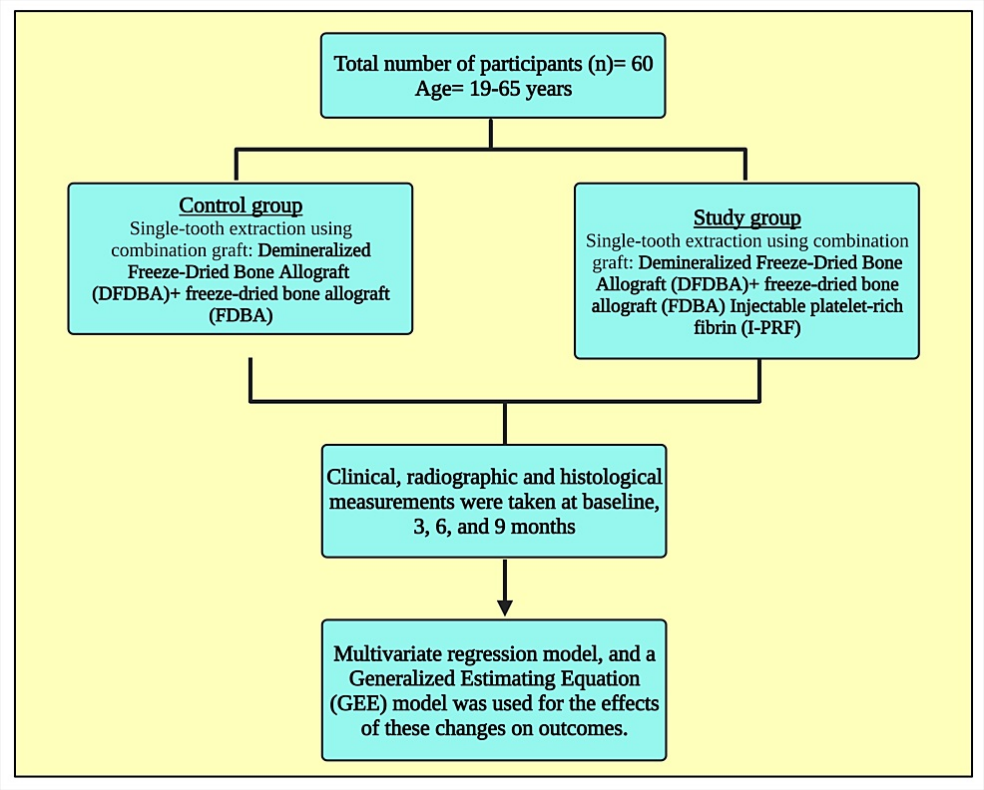
A flow chart portraying the study design Notes: This figure has been drawn with the premium version of BioRender (https://biorender.com/ accessed on September 23, 2023) with the license number LI25VX6EWO. Image credit: Susmita Sinha PRF: platelet-rich fibrin; I-PRF: injectable platelet-rich fibrin; DFDBA: demineralized freeze-dried bone allograft; FDBA: freeze-dried bone allograft

This was a clinical, radiographic, and histological study conducted in the Department of Periodontics at Karnavati School of Dentistry, Gandhinagar, Gujarat, India. Prior permission and consent were obtained from the patients. After considering all the inclusion and exclusion criteria, 60 participants were selected for the study, aged between 19 and 65 (Figure 1).

Sampling method

Sixty patients were selected without maintaining any inequity of socio-economic status, religion, caste, and sex. After obtaining the informed consent, study procedures were performed. The sampling method used was simple random sampling in which subjects were divided randomly into two groups (control and test group) by coin toss method. The sample size was calculated by using the following formula: n= (Zα/2 + Zβ)2 *2* σ2/d2, where Zα/2 = the critical value of the normal distribution at α/2 (confidence level = 95%, α = 0.05, and the critical value = 1.96), Zβ = the critical value of the normal distribution at β (power = 80%, β = 0.2 and the critical value = 0.84), σ2 = population variance, and d = the difference researcher would like to detect [78].

Patient selection principles

Inclusion Criteria

Patients aged between 18 and 65 years who required implants in the mandibular posterior region, who voluntarily agreed to avoid any fixed prosthesis for nine months, and who gave written consent for the therapeutic intervention were included in this research.

Exclusion Criteria

Patients with poor oral hygiene, compromised medical conditions such as diabetes and heart-related disease, on medication that could alter the restoration of periodontal trauma such as corticosteroids and Ca2+ channel blockers, with medication reactions, with a history of chronic illness, pregnant females, teeth indicated for extraction for orthodontic purposes, third molar teeth indicated for extraction, and with history of smoking were excluded from the study.

Surgical methods

The dental surgical process was done under local anaesthesia, using 2% lignocaine hydrochloride with adrenaline as 1:80,000. According to the technique developed by Miron et al. [79] for the formulating of I-PRF, 10 ml of whole blood was taken from the experiment group patient without anticoagulant in plastic tubes and centrifuged at 700 rpm for three minutes (60×g) at standard temperature (23°C or 73.4 °F) and pressure in a centrifuge machine. The topmost liquescent layer was collected as I-PRF. In both groups, atraumatic extraction was performed gently to not damage the alveolar ridge. The buccolingual width measurement was taken using a vernier calliper. A radiograph at baseline was taken after extraction. Bone graft DFDBA (500-1040 μm) and FDBA (500-1040 μm) were mixed in a 30:70 ratio for the control group. The dental defect (socket formation) was shielded with a bio-resorbable collagen plug to protect the remaining 1-2 mm augmented socket over graft material, and then a 3-0 non-recordable silk figure-of-eight suture was taken for retention of graft material in the dental extraction pocket and roofed with periodontal pack. In the test group, bone graft DFDBA and FDBA were mixed in a 30:70 ratio, and the I-PRF was mixed with bone graft left for five to seven minutes for polymerization to form the sticky bone. The mix was then placed into the dental extraction socket. A collagen plug was placed to cover the tenacious bone and then sutured to the adjacent soft tissues using non-resorbable suturing material and a periodontal pack like the control group.

The patient remains on antimicrobial medication for three days, accompanied by anodynes for three days. Chlorhexidine gluconate solution (0.2% of 5-10 ml to be rinsed for five minutes twice daily) was instituted for the first postoperative week. After 10 days, patients were summoned back for removal of the black silk sutures, evaluation of the tissue reaction, and recovery from the surgical wound. Radiographic and clinical parameters were appraised at baseline, three months, six months, and nine months. After nine months, after taking the radiograph, local anaesthesia was given to the patient, crestal incision was granted, and a flap was reflected. Then, the ridge was measured using a vernier calliper.

A bone biopsy was sent to the Karnavati School of Dentistry's Oral Pathology Department for histological evaluation. At the time of osteotomy preparation, a bone slice was obtained using a trephine bur for histological processing before the implant was inserted. The sample was then transferred into 10% neutral buffered formalin (fixative) for 24 hours. Then, the tissue sample was placed in a 5% nitric acid solution for more than 48 hours for decalcification. The solution was changed every 24 hours, then dehydrated in ethanol and embedded in paraffin. When decalcification was completed, the tissue sample was rinsed with water to remove acid particles. The sample was transferred into an automatic tissue processor for tissue processing and followed by a routine hematoxylin and eosin staining procedure.

The histomorphometric analysis was carried out for each core; a few sections were created. Each part was magnified four times to see which produced the most outstanding high-power evaluation. The portion was analyzed at a magnification of 10-40x to safeguard precise investigation of new vital bone, residual graft, and connective tissue/other. The connective tissue/other group includes vascularity, loose fibrous connective tissue, and inflammatory cells. Researchers utilized image editing software to confirm after the core was segmented into manifold constituents (residual graft, vital bone, connective tissue, etc.). To establish the percent area of each element of the bone core, the total number of pixels for each picture was computed and counted, and the percentage of pixels for each image was determined.

Landry et al. first coined the healing index [80-84] to portray the level of clinical healing following periodontal surgery, and it was updated to be utilized for extraction socket healing after 10 days. Radiovisiographs (RVG) were obtained utilizing the Rinn XCP system (Dentsply Sirona, Charlotte, North Carolina, United States) and a uniform paralleling approach using a typical intraoral grid. To assess the alveolar bone height, the radiographic data were recorded with a grid at the preoperative baseline, three months, six months, and nine months after surgery. 

Ethical approval

The Ethics Committee of the Karnavati School of Dentistry, Karnavati University, Gandhinagar, Gujarat, India, approved this study (approval number: KSDEC/20-21/Apr/09, dated December 18, 2020). All study subjects were briefed regarding the aims and objectives of the study and future publication. Written informant consent was obtained before any intervention was conducted. Additionally, this study was anonymous, and research participants had every right to refuse to be included.

Statistical analysis

A multivariate regression model was employed to track alterations in the area of defect, percent bone filling, linear bone growth, and buccolingual width as dependent variables. The two groups (test and control) were utilized as independent variables to explore changes in outcome levels at baseline, three months, six months, and nine months. The multivariate regression analysis was adjusted for age and sex. Patients were repeatedly assessed with time, spanning from baseline through three, six, and nine months. A generalized estimating equation (GEE) model utilizing an exchangeable correlation matrix was employed to evaluate the effects of these changes on outcomes. The statistical models were adjusted for age, sex, time points (baseline, three months, six months, and nine months), and the interaction between the treatment group and time. A p-value ≤ 0.05 was considered as significant. Data was analyzed using the Stata Statistical Software: Release 15 (2017; StataCorp LLC, College Station, Texas, United States), and GraphPad Prism 8.3.0 (2019; Dotmatics, Boston, Massachusetts, United States) was used for the graphical presentation. 

## Results

**Figure 2:**
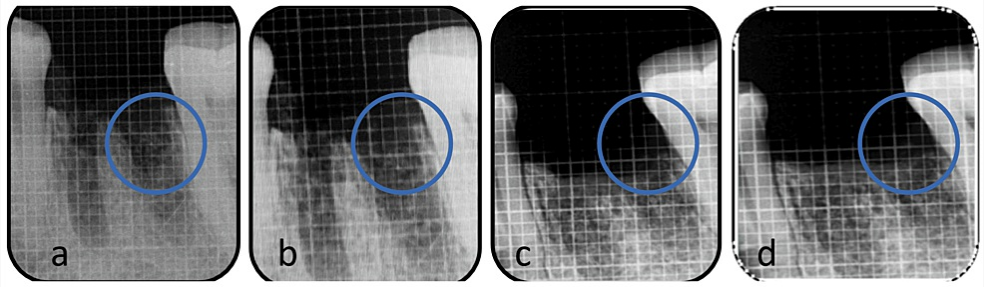
The areas marked in blue circle show: (a) the extracted socket at baseline, (b) mild bone formation at the end of three months, (c) moderate bone formation at six months, and (d) even denser bone at nine months, which signifies constant bone formation.

**Figure 3:**
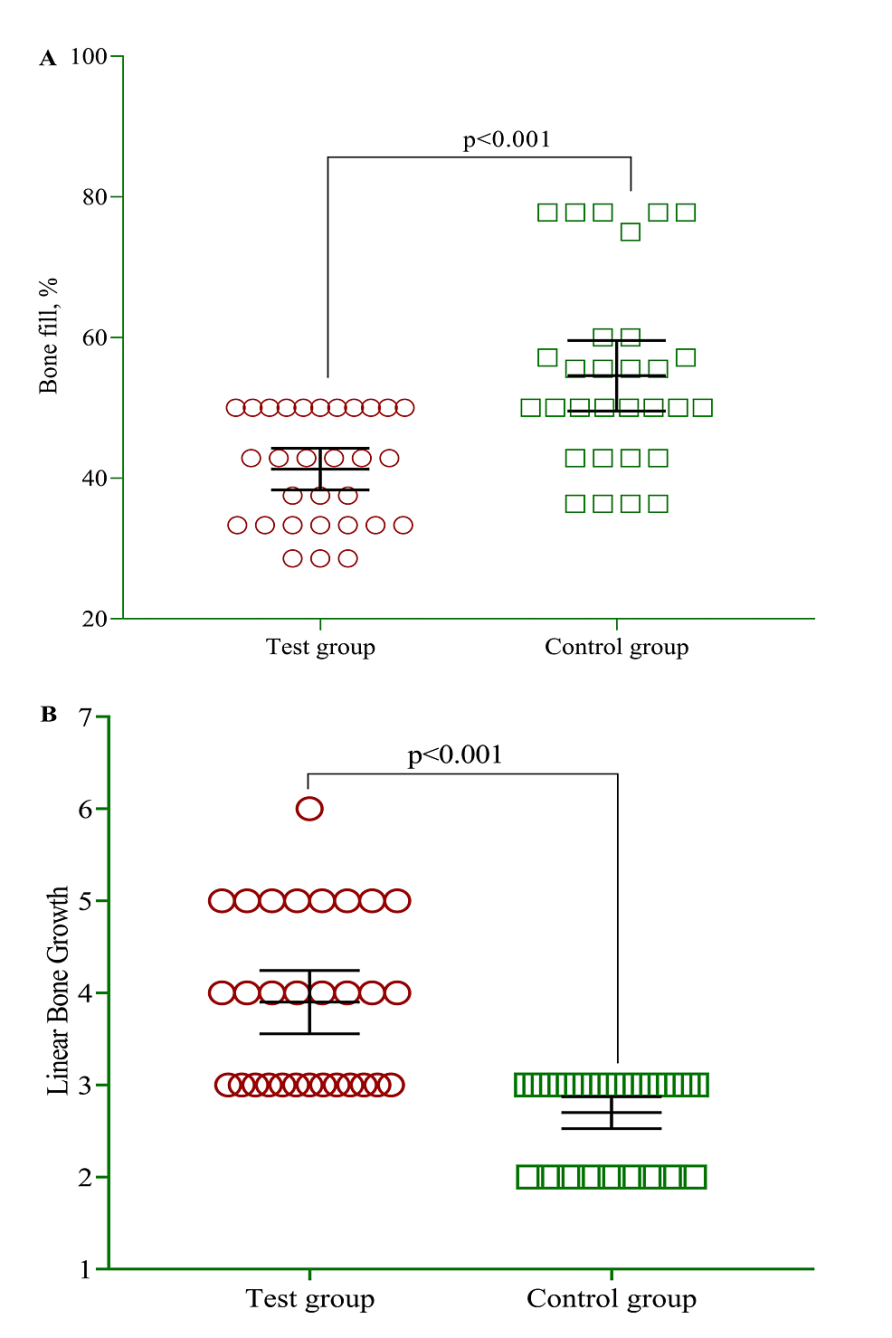
Mean difference of percent bone fill and linear bone growth between test and control group. The p-value was estimated using a multivariate regression model adjusted for age and sex

The measurement of crestal bone height was conducted radiographically, employing the extended cone technique and RVG technology. To establish a standardized radiograph, a radiopaque millimetre-graduated grid was used with a radiographic imaging technique known as RVG. This approach was adopted to mitigate potential distortions and facilitate accurate measurements of the crestal bone height, as depicted in Figure 2. The spatial separation amid the two radiopaque squares within the grid was measured to be 1 mm. The areas of defect are filled with new bone, which was visible on radiographs taken at baseline (Figure 3a), three months (Figure 3b), six months (Figure 3c), and nine months (Figure 3d).

When analyzing the detectable area between the test and control groups during different study periods, a notable increase was identified at baseline (β=7.14, 95%CI= -0.88, -15.2, p=0.050). In contrast, a significant decline was noted at the nine-month mark (β=-2.94, 95%CI= -8.29, -0.30, p=0.044) compared to the investigated group, as outlined in Table 1. Given the significant difference observed at baseline, the GEE model was further adjusted to include baseline as a covariate. This adjustment revealed a noteworthy decrease in the test group (β=-0.98, 95%CI= -5.11, -0.04, p=0.031) than in the control group (Table 1).

**Table 1:** Association between the experimental batch and the control group around defect at baseline, three months, six months, nine months, and overall changes in the test cluster Notes: The p-value was estimated using a multivariate regression model, adjusted for age and sex. *The study employed a GEE model with an exchangeable correlation matrix to examine the overall modifications between the experimental folk and the control sample model. The statistical model was adjusted for covariates, including age, sex, and time points (Baseline,  three months, six months, nine months). Furthermore, the data was corrected based on the established baseline. GEE: generalized estimating equation

	β (95% CI)	p-value
Area of defect		
Baseline	7.14(0.88, 15.20)	0.050
Month 3	-0.05(-7.18, 7.09)	0.990
Month 6	-0.23(-6.73, 6.27)	0.943
Month 9	-2.94(-8.19, -0.3)	0.044
^*^Overall changes	0.67(-5.11, 7.07)	0.753
^#^Overall changes	-0.98(-5.11, -0.04)	0.031

In Figure 2 and Figure 3A, the test group displayed a significantly more pronounced level of bone filling as a proportion (54.6±13.4), in contrast to the control group (41.3±7.92), indicating a substantial increase (p<0.001). Furthermore, in Figure 3B, the study findings revealed that the experimental cluster had a significantly greater linear bone development (3.90±0.92) than the control group (2.70±0.47). Despite considering age and sex as covariates during the analysis, no discernible impact modification was identified.

**Figure 4:**
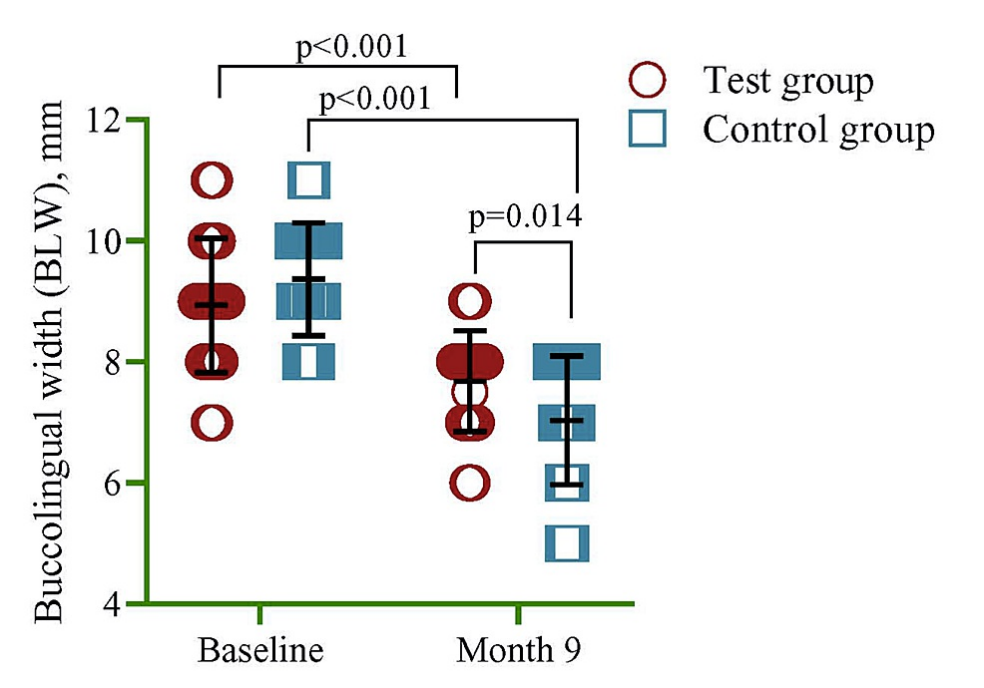
Comparison of buccolingual between test and control group at baseline and at six months. The researchers employed a multivariate regression model to assess the comparability between groups, while a repeated measure ANOVA was used to examine the difference between groups at baseline and nine months. The model was adjusted for age and sex.

The buccolingual thickness of the alveolar bone was quantified in mm using vernier callipers at the crestal level. Researchers employed a repeated measure analysis of variance (ANOVA). It was ascertained that the buccolingual width exhibited a statistically significant decrease (p<0.001) at the nine-month time point compared to the baseline for both the control and investigated clusters. Nevertheless, the findings derived from a multivariate regression analysis revealed that, following a period of nine months, the experimental group (mean=7.68, SD=0.84) displayed a statistically noteworthy rise in buccolingual width equated to the control assemblage (mean=7.03, SD=1.07), as depicted in Figure 4.

**Figure 5:**
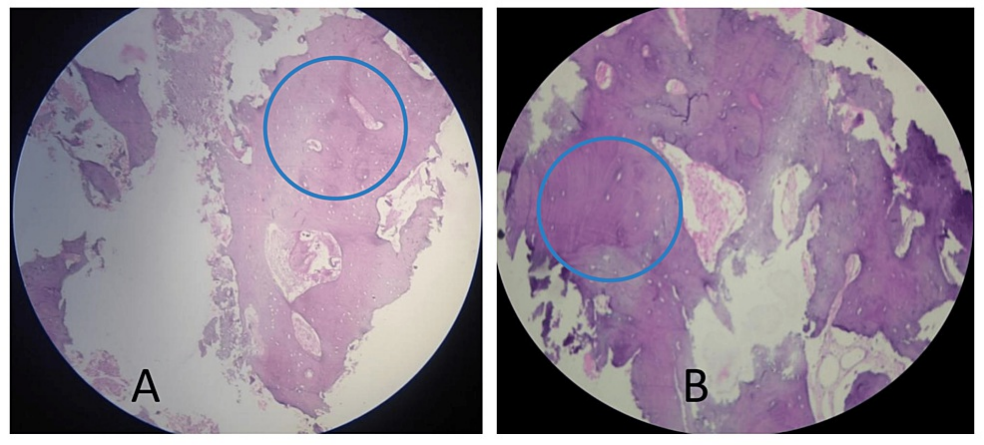
The histological sections of the control group (A) and the experimental group (B) show small areas of newly formed bone with plump osteocytes inside lacunae, also seen mostly in peripheral lamellar bone surrounding the residual graft material. The blue circles signify osteocytes residing within lacunae, which are recognized as essential bone components, while absence of osteocytes indicate a residual graft.

In this study of histological specimens, both groups utilized IMAGE J software (National Institutes of Health, Stapleton, New York, United States) to quantify the proportions of new bone, provisional matrix, and residual graft (Figure 5). The test group exhibited a notably more significant percentage of vital bone than the control group, with a p-value to a lesser extent than 0.001 (Table 2). This finding was consistent with the observation in other instances, where the test group consistently demonstrated a more significant percentage than the control assembly, with a p-value of less than 0.001 (Figure 5).

**Table 2:** Mean difference of percent vital bone, residual graft, and others between test and control group Notes: Data was presented as mean±SD, and an independent sample t-test was used to estimate the p-value

	Control group (n=30)	Test group (n=30)	p-value
Vital bone, %	38.4±12.5	42.6±1.76	0.076
Residual graft, %	14.3±6.80	7.69±1.78	<0.001
Others, %	52.5±7.97	43.9±1.03	<0.001

## Discussion

The principal objective of the current study was to assess bone repair at the socket site after extraction utilizing bone graft (DFDBA and FDBA) and I-PRF. Chenchev et al. employed blended bone graft materials and PRF in their study, and the observed positive clinical and radiographic outcomes suggest potential benefits in utilizing advanced PRF (A-PRF) and I-PRF for alveolar ridge bone augmentation before implant placement [85]. Autogenous bone grafts are challenging to harvest [85] and require a second surgical site. The utilization of the combination allograft stemmed in a statistically suggestive escalation in the average amount of viable bone development and a decrease in the remaining amount of non-viable material. Harvesting autogenous bone grafts poses substantial challenges [86-88] and necessitates a second surgical site [89,90]. At 18-20 weeks after succeeding alveolar ridge perpetuation, the utilization of a combination allograft yielded a significantly greater average formation of vivacious osseous matter and reduced the number of transplanted substances [91-93]. When assessing the capacity to create essential bone in alveolar ridge preservation, DFDBA alone has been demonstrated to be grander for spontaneous therapeutic processes [94,95].

On the other hand, using FDBA as an osteoconductive platform for new bone construction enables the preservation of airspace and clot stability throughout the healing process [64,96,97]. Forty-two individuals were assigned randomly, resulting in two groups of identical size. These groups were administered 100% mineralized FDBA (active control group) or a 70% mineralized:30% demineralized allograft (experimental cluster) for ridge conservancy. This study supports with histologic evidence that a mix of mineralized/demineralized allograft results in higher new bone growth compared to 100% mineralized FDBA in alveolar ridge preservation in people. This research results were in the same line with earlier research reports [91,98]. Paralleled to FDBA unaided in alveolar ridge salvation, a combination allograft improves vital bone development while offering equivalent structural permanence of the alveolar crest [91,99]. Thus, we have used a combination graft here. No earlier study has previously compared socket site preservation using bone graft and I-PRF in India. Therefore, the present analysis evaluated the socket site preservation succeeding single tooth extraction using a combination of composite grafts alone and I-PRF in bone rejuvenation when placed in the extraction socket.

No statistically noteworthy dissimilarity was detected amid the two assemblages, suggesting no significant disparities in the healing of wound sockets. This lack of difference may be attributed to the utilization of collagen plugs and the suturing location in both the experimental and control sites. Additionally, selecting patients free from periodontal or asymptomatic apical periodontitis in mutually investigated and control sites may have contributed to these findings [14].

The alveolar bone width was measured at the crestal level using vernier callipers, with the unit of measure being mm [100]. However, other studies reported that an innovative cone-beam computed tomography (CBCT) scanning procedure is preferred [101,102]. The test group exhibited a greater buccolingual width than the control group. Only crestal bone levels are measured because crestal alveolar bone is most altered. According to a study by Nisaret al., the crestal levels showed maximum alterations from baseline to six months [14]. Any benefit in new bone growth from the resorbable collagen dressing could be due to the graft substance. This was significant because one of potential PRF's benefits is its thick fibrin arrangement, which may have cell plosive potentials.

On the other hand, the absence of a membrane could be problematic [99,103]. Famili et al. studied 20 extraction sockets in a sample of six persons [75]. The alveolar elevation was continued using a DFDBA or a 70:30 mixed allograft. The results showed minimal differences between the two grafting ingredients when utilizing CBCT to measure deviations in the alveolar ridge features.

Wang et al. studied using I-PRF instead of standard PRP improved osteoblast behaviour [104]. Based on the researchers' findings, it was seen that the administration of PRP resulted in a two-fold higher in the migration of osteoblastic cells than to the control faction. The migration rate of I-PRF was three times higher than that of tissue culture plastic. In contrast to PRP, the researchers observed that I-PR exhibits a notably elevated proliferation rate for three to five days. According to their assertion, the utilization of I-PRF yielded more reliable results. I-PRF has been observed to induce a significant enhancement in cellular migration. The messenger RNA levels of PDGF, TGF-, type I collagen, and fibronectin were elevated in I-PRF than in PRP. The researchers concluded that I-PRF utilization is crucial and can be achieved without anticoagulants.

Thanasrisuebwong et al. employed red I-PRF, utilizing the top yellow zone for the collection of yellow I-PRF and both the yellow and red zones of the buffy coat for the collection of red I-PRF fractions [105]. Based on the findings of the scientists, it was observed that red I-PRF exhibited enhanced biological capabilities and demonstrated the release of growth factors during a timeframe of 7-14 days. Furthermore, it has been observed that the yellow I-PRF exhibits enhanced viscoelastic characteristics.

In a recent study conducted by Kyyak et al., the researchers examined the impact of two different bone replacement materials, namely allogenic bone replacement material (ABSM) and xenogenic bone replacement material (XBSM), on the cellular characteristics of human osteoblasts [106]. The examination also explored the impacts of incorporating an additional element known as I-PRF into both materials. The study employed an in vitro experimental design [106]. I-PRF has been observed to enhance cellular migration proliferation differentiation when used independently and, to a lesser extent, when combined with ABSM and XBSM. This phenomenon can potentially confer a clinical benefit in accelerated bone repair. Gülşen et al. appraised the process of new bone formation after sinus floor augmentation. This investigation specifically focused on utilizing collagen plugs as carriers for I-PRF research [107]. A notable disparity in the construction of new bone was seen during the six-month evaluation period. Sinus floor augmentation involved the regeneration of new bone using I-PRF, which was delivered via collagen plugs. Several studies have indicated that non-restorable molar teeth, which had undergone endodontic treatment, underwent expedited bone grafting to facilitate subsequent implant placement. The application of I-PRF was employed to enhance the efficacy of each bone graft, leading to a notable increase in the proportion of newly formed essential bone [108-110]. This is likely due to the constant I- PRF's gradual release and supply of growth factors [111,112].

Results of the current study indicated bone growth with increasing time intervals for both groups from baseline to nine months but they were better in the experimental group than in the control. Nisar et al. showed improvement in linear bone growth for both groups compared to baseline [14]. In our study, the improvement in the test group using bone graft with combination allograft showed promising results compared to combination bone allograft used alone. Linear bone growth showed a significant difference between both groups, and the amount of bone growth in the test cluster was higher than in the control group. Also, the bone fill percent was 54% in the experimental group and 41% in the control group. Among all the parameters taken, the test group indicated good bone growth with increasing intervals when the socket site was grafted with bone graft in combination with I-PRF.

The study data was obtained radiographically using a grid, followed by Thakkar et al. [113]; vernier callipers were used to measure the breadth of the alveolar ridge, and a grid was used to calibrate radiographs. When both groups were evaluated, the findings of this study revealed a substantial drop in ridge width and height for both groups after 90 and 180 days. PRF paired with DFDBA retained ridge width more effectively than DFDBA alone. Consequently, radiographic parameters are standardized when employing a calibrated grid.

In the analysis of histological specimens, both groups utilized ImageJ software, a publicly available Java image processing program developed at the National Institutes of Health and the Laboratory for Optical and Computational Instrumentation (LOCI), University of Wisconsin, Madison, Wisconsin, United States, to measure the proportions of new bone, provisional matrix, and residual graft. The control group exhibited a mean value of percent vital bone at 38.4±12.5, while the test group displayed a mean value of 42.6±1.76. This disparity between the two groups was found to be statistically significant. The percentage of residual graft also exhibited a considerable gap, with a lower value observed in the test group compared to the control group. In contrast, the two groups observed no statistically significant differences in other inflammatory cells and connective tissue. The extent of crucial bone growth observed following ridge preservation exhibits significant variability, contingent upon the specific grafting material employed. In a study conducted by Clark *et al*., (2018), the researchers examined the utilization of autologous PRF in combination with FDBA and FDBA or A-PRF alone [99]. Based on the research findings, the variety of FDBA with A-PRF demonstrated superior histological outcomes.

**Figure 6:**
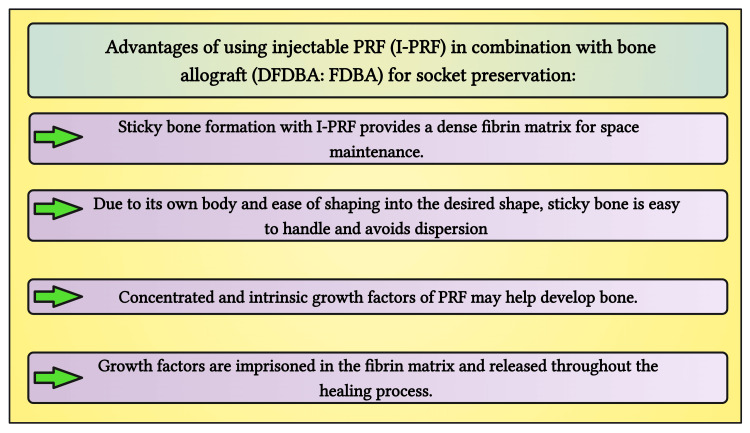
Chart showing advantages of injectable PRF (I-PRF) combined with bone allograft (DFDBA: FDBA) for socket preservation Notes: This figure has been drawn with the premium version of BioRender (https://biorender.com/ accessed on September 23, 2023) with the license OH25VXE474. Image credit: Susmita Sinha PRF: platelet-rich fibrin; I-PRF: injectable platelet-rich fibrin; DFDBA: demineralized freeze-dried bone allograft; FDBA: freeze-dried bone allograft

**Figure 7:**
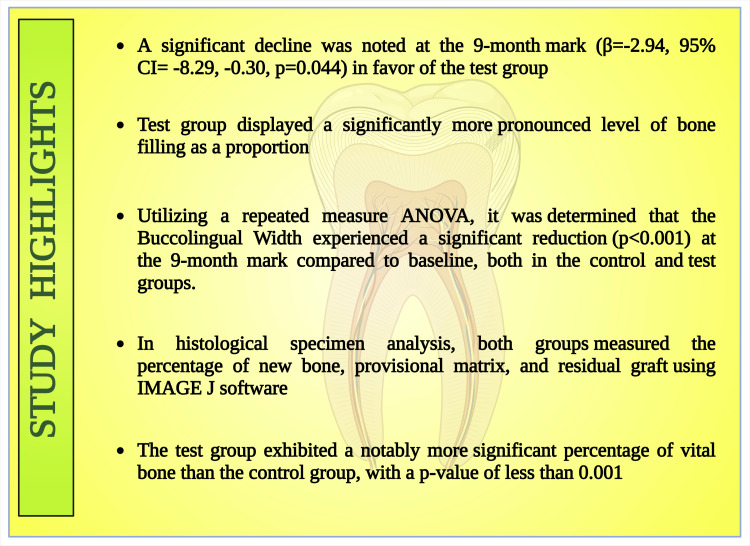
A chart showing the study highlights Notes: This figure has been drawn with the premium version of BioRender (https://biorender.com/ accessed on September 23, 2023) with the license number GO25VXAS23. Image credit: Susmita Sinha

Sticky bone formation with I-PRF provides a dense fibrin matrix for space maintenance. Due to its own body and ease of shaping into the desired shape, sticky bone is easy to handle and avoids dispersion [114]. Though using only DFDBA and FDBA in 30, a 70 ratio, Borg and Mealey conducted another study that reported more residual graft and less vital bone content [91]. The DFDBA:FDBA treated sites' slower essential bone formation might have been caused by mineralized graft material. A graft material that slowly dissolves might be advantageous since it keeps space during healing. However, neovascularization and new tissue ingrowth are necessary for the graft material-filled wound to heal correctly. Adding graft material like FDBA to the mix will likely suppress osteogenesis by delaying healing or lengthening resorption. A-PRF's concentrated and intrinsic growth factors may help develop bone. Growth factors are sequestered inside the fibrin mesh and liberated throughout the recovery phase (Figure 6). This pledges that growth factors are delivered at the appropriate moment to stimulate bone development in a concentrated and sustained way. Figure 7 illustrates the highlights of this study.

Limitations of this study

A large sample size would have been preferable with a longer follow-up duration. Moreover, a vernier calliper was employed in lieu of a manual calliper, with the option of utilizing a digital calliper. In this investigation, clinical assessments were conducted in the absence of stents. The accuracy of the ridge width and ridge height measurements obtained during extraction, compared to those taken after nine months, may have been compromised due to the absence of precise and fixed reference points. In this study, we utilized a graft consisting of DFDBA and FDBA in a ratio of 30:70. It is worth noting that this graft ratio lacks substantial literature support. Additionally, CBCT was employed to enhance the accuracy of bone fill estimation. One other limitation of the study was the absence of demographic information; however, we did collect data on comorbidities that could potentially impact the outcomes. 

## Conclusions

The materials utilized in this investigation produced favorable outcomes for preserving bone height but no appreciable effects for maintaining bone breadth. Additionally, histological evidence shows that using bone graft and I-PRF together results in higher bone height and higher percentages of new vital bone compared to using bone graft alone. To sum up, a predictable choice for socket grafting is I-PRF with bone transplant. The findings of this study determine the recreating properties of I-PRF at a previously healed extraction site, suggesting its potential usages beyond ridge preservation. To ascertain the ridge preservation capabilities of these materials, future research endeavors must extend the evaluation of the osteogenic prospect of I-PRF to encompass more comprehensive ridge amplification techniques. Additionally, there is a need to explore the regenerative capacities of I-PRF in the context of gum disease (pyorrhea, periodontitis) regarding regeneration and to conduct in vivo analyses with a nobler sample magnitude.

## References

[1] Avila-Ortiz G, Elangovan S, Kramer KW, Blanchette D, Dawson DV (2014). Effect of alveolar ridge preservation after tooth extraction: a systematic review and meta-analysis. J Dent Res.

[2] Avila-Ortiz G, Chambrone L, Vignoletti F (2019). Effect of alveolar ridge preservation interventions following tooth extraction: a systematic review and meta-analysis. J Clin Periodontol.

[3] Atieh MA, Alnaqbi M, Abdunabi F, Lin L, Alsabeeha NH (2022). Alveolar ridge preservation in extraction sockets of periodontally compromised teeth: a systematic review and meta-analysis. Clin Oral Implants Res.

[4] El-Sioufi I, Oikonomou I, Koletsi D, Bobetsis YA, Madianos PN, Vassilopoulos S (2023). Clinical evaluation of different alveolar ridge preservation techniques after tooth extraction: a randomized clinical trial. Clin Oral Investig.

[5] Moreno AR, Magdaleno MO, Islas MM, Mercado JA, Del Pilar Goldaracena Azuara M, Cruz ER, Ramírez GF (2019). Postextraction alveolar preservation and use of the crown of the extracted tooth as a temporary restoration. Case Rep Dent.

[6] Chisci G, Hatia A, Chisci E, Chisci D, Gennaro P, Gabriele G (2023). Socket preservation after tooth extraction: particulate autologous bone vs. deproteinized bovine bone. Bioengineering (Basel).

[7] Ucer C, Khan RS (2023). Extraction socket augmentation with autologous platelet-rich fibrin (PRF): the rationale for socket augmentation. Dent J (Basel).

[8] Heimes D, Schiegnitz E, Kuchen R, Kämmerer PW, Al-Nawas B (2021). Buccal bone thickness in anterior and posterior teeth-a systematic review. Healthcare (Basel).

[9] Sáez-Alcaide LM, González Fernández-Tresguerres F, Cortés-Bretón Brinkmann J (2021). Socket shield technique: a systematic review of human studies. Ann Anat.

[10] Passarelli PC, Pagnoni S, Piccirillo GB (2020). Reasons for tooth extractions and related risk factors in adult patients: a cohort study. Int J Environ Res Public Health.

[11] Pagni G, Pellegrini G, Giannobile WV, Rasperini G (2012). Postextraction alveolar ridge preservation: biological basis and treatments. Int J Dent.

[12] Hansson S, Halldin A (2012). Alveolar ridge resorption after tooth extraction: a consequence of a fundamental principle of bone physiology. J Dent Biomech.

[13] Steigmann L, Di Gianfilippo R, Steigmann M, Wang HL (2022). Classification based on extraction socket buccal bone morphology and related treatment decision tree. Materials (Basel).

[14] Nisar N, Nilesh K, Parkar MI, Punde P (2020). Extraction socket preservation using a collagen plug combined with platelet-rich plasma (PRP): a comparative clinico-radiographic study. J Dent Res Dent Clin Dent Prospects.

[15] Kotsakis G, Chrepa V, Marcou N, Prasad H, Hinrichs J (2014). Flapless alveolar ridge preservation utilizing the "socket-plug" technique: clinical technique and review of the literature. J Oral Implantol.

[16] Avila-Ortiz G, Rodriguez JC, Rudek I, Benavides E, Rios H, Wang HL (2014). Effectiveness of three different alveolar ridge preservation techniques: a pilot randomized controlled trial. Int J Periodontics Restorative Dent.

[17] Sun Y, Wang CY, Wang ZY, Cui Y, Qiu ZY, Song TX, Cui FZ (2016). Test in canine extraction site preservations by using mineralized collagen plug with or without membrane. J Biomater Appl.

[18] Jambhekar S, Kernen F, Bidra AS (2015). Clinical and histologic outcomes of socket grafting after flapless tooth extraction: a systematic review of randomized controlled clinical trials. J Prosthet Dent.

[19] Omi M, Mishina Y (2022). Roles of osteoclasts in alveolar bone remodeling. Genesis.

[20] Kondo T, Kanayama K, Egusa H, Nishimura I (2023). Current perspectives of residual ridge resorption: pathological activation of oral barrier osteoclasts. J Prosthodont Res.

[21] Tsuchida S, Nakayama T (2023). Recent clinical treatment and basic research on the alveolar bone. Biomedicines.

[22] Farmer M, Darby I (2014). Ridge dimensional changes following single-tooth extraction in the aesthetic zone. Clin Oral Implants Res.

[23] Couso-Queiruga E, Stuhr S, Tattan M, Chambrone L, Avila-Ortiz G (2021). Post-extraction dimensional changes: a systematic review and meta-analysis. J Clin Periodontol.

[24] Levi I, Halperin-Sternfeld M, Horwitz J, Zigdon-Giladi H, Machtei EE (2017). Dimensional changes of the maxillary sinus following tooth extraction in the posterior maxilla with and without socket preservation. Clin Implant Dent Relat Res.

[25] Kim YK, Ku JK (2020). Extraction socket preservation. J Korean Assoc Oral Maxillofac Surg.

[26] Dayakar MM, Waheed A, Bhat HS, Gurpur PP (2018). The socket-shield technique and immediate implant placement. J Indian Soc Periodontol.

[27] Kim JJ, Ben Amara H, Chung I, Koo KT (2021). Compromised extraction sockets: a new classification and prevalence involving both soft and hard tissue loss. J Periodontal Implant Sci.

[28] Leblebicioglu B, Salas M, Ort Y (2013). Determinants of alveolar ridge preservation differ by anatomic location. J Clin Periodontol.

[29] Zhao L, Wei Y, Xu T, Zhang B, Hu W, Chung KH (2019). Changes in alveolar process dimensions following extraction of molars with advanced periodontal disease: a clinical pilot study. Clin Oral Implants Res.

[30] Jamjoom A, Cohen RE (2015). Grafts for ridge preservation. J Funct Biomater.

[31] Mehta H, Shah S (2015). Management of buccal gap and resorption of buccal plate in immediate implant placement: a clinical case report. J Int Oral Health.

[32] Hu L, Han X, Zhang D, Wu J, Huang S (2023). Buccal plate preservation with immediate post-extraction implant placement and provisionalization in anterior maxillary tooth: preliminary results of a new technique using Teruplug collagen. J Stomatol Oral Maxillofac Surg.

[33] Guruprasad Y, Dhurubatha J, Kumar S, Sultana R, Bakshi HT, Desai DT (2023). A comparative study of the flap and flapless techniques of ridge preservation: a clinical double-blinded study. J Pharm Bioallied Sci.

[34] Maier FM (2016). Initial crestal bone loss after implant placement with flapped or flapless surgery-a prospective cohort study. Int J Oral Maxillofac Implants.

[35] Siu TL, Dukka H, Saleh MH (2023). Flap versus flapless alveolar ridge preservation: a clinical and histological single-blinded, randomized controlled trial. J Periodontol.

[36] Alfailany DT, Hajeer MY, Burhan AS, Mahaini L, Darwich K, Aljabban O (2022). Evaluation of the effectiveness of surgical interventions versus non-surgical ones when used in conjunction with fixed appliances to accelerate orthodontic tooth movement: a systematic review. Cureus.

[37] Uppala S, Parihar AS, Modipalle V, Manual L, Oommen VM, Karadiguddi P, Gupta P (2020). Crestal bone loss around dental implants after implantation of tricalcium phosphate and platelet- rich plasma: a comparative study. J Family Med Prim Care.

[38] Tadi DP, Pinisetti S, Gujjalapudi M, Kakaraparthi S, Kolasani B, Vadapalli SH (2014). Evaluation of initial stability and crestal bone loss in immediate implant placement: an in vivo study. J Int Soc Prev Community Dent.

[39] Arshad S, Tehreem F, Rehab Khan M, Ahmed F, Marya A, Karobari MI (2021). Platelet-rich fibrin used in regenerative endodontics and dentistry: current uses, limitations, and future recommendations for application. Int J Dent.

[40] Pavlovic V, Ciric M, Jovanovic V, Trandafilovic M, Stojanovic P (2021). Platelet-rich fibrin: basics of biological actions and protocol modifications. Open Med (Wars).

[41] Grecu AF, Reclaru L, Ardelean LC, Nica O, Ciucă EM, Ciurea ME (2019). Platelet-rich fibrin and its emerging therapeutic benefits for musculoskeletal injury treatment. Medicina (Kaunas).

[42] Verma UP, Yadav RK, Dixit M, Gupta A (2017). Platelet-rich fibrin: a paradigm in periodontal therapy - a systematic review. J Int Soc Prev Community Dent.

[43] Al-Maawi S, Becker K, Schwarz F, Sader R, Ghanaati S (2021). Efficacy of platelet-rich fibrin in promoting the healing of extraction sockets: a systematic review. Int J Implant Dent.

[44] Farshidfar N, Jafarpour D, Firoozi P, Sahmeddini S, Hamedani S, de Souza RF, Tayebi L (2022). The application of injectable platelet-rich fibrin in regenerative dentistry: a systematic scoping review of In vitro and In vivo studies. Jpn Dent Sci Rev.

[45] Alan R, Ercan E, Firatli Y, Firatli E, Tunali M (2023). Innovative i-PRF semi-surgical method for gingival augmentation and root coverage in thin periodontal phenotypes: a preliminary study. Quintessence Int.

[46] Reksodiputro MH, Harahap AR, Setiawan L, Yosia M (2021). A modified preparation method of ideal platelet-rich fibrin matrix from whole blood. Front Med (Lausanne).

[47] Karimi F, Biazar E, Heidari-Keshel S, Pourjabbar B, Khataminezhad MR, Shirinbakhsh S, Zolfaghari-Moghaddam SY (2022). Platelet-rich fibrin (PRF) gel modified by a carbodiimide crosslinker for tissue regeneration. RSC Adv.

[48] Gollapudi M, Bajaj P, Oza RR (2022). Injectable platelet-rich fibrin - a revolution in periodontal regeneration. Cureus.

[49] Dashore S, Chouhan K, Nanda S, Sharma A (2021). Platelet-rich fibrin, preparation and use in dermatology. Indian Dermatol Online J.

[50] Shashank B, Bhushan M (2021). Injectable platelet-rich fibrin (PRF): the newest biomaterial and its use in various dermatological conditions in our practice: a case series. J Cosmet Dermatol.

[51] van Orten A, Goetz W, Bilhan H (2022). Tooth-derived granules in combination with platelet-rich fibrin ("sticky tooth") in socket preservation: a histological evaluation. Dent J (Basel).

[52] Pall E, Roman A, Olah D, Beteg FI, Cenariu M, Spînu M (2023). Enhanced bioactive potential of functionalized injectable platelet-rich plasma. Molecules.

[53] Kökdere NN, Baykul T, Findik Y (2015). The use of platelet-rich fibrin (PRF) and PRF-mixed particulated autogenous bone graft in the treatment of bone defects: an experimental and histomorphometrical study. Dent Res J (Isfahan).

[54] Liu Y, Sun X, Yu J (2019). Platelet-rich fibrin as a bone graft material in oral and maxillofacial bone regeneration: classification and summary for better application. Biomed Res Int.

[55] Kızıldağ A, Tasdemir U, Arabacı T, Kızıldağ CA, Albayrak M, Şahin B (2020). Effects of autogenous tooth bone graft and platelet-rich fibrin in peri-implant defects: an experimental study in an animal model. J Oral Implantol.

[56] Karayürek F, Kadiroğlu ET, Nergiz Y, Coşkun Akçay N, Tunik S, Ersöz Kanay B, Uysal E (2019). Combining platelet rich fibrin with different bone graft materials: an experimental study on the histopathological and immunohistochemical aspects of bone healing. J Craniomaxillofac Surg.

[57] Idiri K, Bandiaky O, Soueidan A, Verner C, Renard E, Struillou X (2023). The effectiveness of the addition of platelet-rich fibrin to bovine xenografts in sinus and bone ridge augmentation: a systematic review. J Funct Biomater.

[58] Almutairi AS (2019). A descriptive analysis of patient’s preferences in bone graft therapy in dentistry. Int J Health Sci (Qassim).

[59] Kumar P, Vinitha B, Fathima G (2013). Bone grafts in dentistry. J Pharm Bioallied Sci.

[60] (2001). Septic arthritis following anterior cruciate ligament reconstruction using tendon allografts--Florida and Louisiana, 2000. MMWR Morb Mortal Wkly Rep.

[61] Langer F, Czitrom A, Pritzker KP, Gross AE (1975). The immunogenicity of fresh and frozen allogeneic bone. J Bone Joint Surg Am.

[62] Friedlaender GE, Strong DM, Sell KW (1976). Studies on the antigenicity of bone. I. Freeze-dried and deep-frozen bone allografts in rabbits. J Bone Joint Surg Am.

[63] Sun X, Liu C, Shi Y, Li C, Sun L, Hou L, Wang X (2019). The assessment of xenogeneic bone immunotoxicity and risk management study. Biomed Eng Online.

[64] Piattelli A, Scarano A, Corigliano M, Piattelli M (1996). Comparison of bone regeneration with the use of mineralized and demineralized freeze-dried bone allografts: a histological and histochemical study in man. Biomaterials.

[65] Atieh MA, Alsabeeha NH, Payne AG, Ali S, Faggion CM, Esposito M (2021). Interventions for replacing missing teeth: alveolar ridge preservation techniques for dental implant site development. Cochrane Database Syst Rev.

[66] Ausenda F, Rasperini G, Acunzo R, Gorbunkova A, Pagni G (2019). New perspectives in the use of biomaterials for periodontal regeneration. Materials (Basel).

[67] Rummelhart JM, Mellonig JT, Gray JL, Towle HJ (1989). A comparison of freeze-dried bone allograft and demineralized freeze-dried bone allograft in human periodontal osseous defects. J Periodontol.

[68] Sheikh Z, Sima C, Glogauer M (2015). Bone replacement materials and techniques used for achieving vertical alveolar bone augmentation. Materials (Basel).

[69] Katuri KK, Kumar PJ, Swarna C, Swamy DN, Arun KV (2013). Evaluation of bioactive glass and demineralized freeze dried bone allograft in the treatment of periodontal intraosseous defects: a comparative clinico-radiographic study. J Indian Soc Periodontol.

[70] Sethi AK, Kar IB, Mohanty T, Mishra N, Singh AK (2018). Use of plasma-enriched demineralized freeze-dried bone matrix in postsurgical jaw defects. Natl J Maxillofac Surg.

[71] Kaigler D, Avila G, Wisner-Lynch L (2011). Platelet-derived growth factor applications in periodontal and peri-implant bone regeneration. Expert Opin Biol Ther.

[72] Reddi AH (1998). Role of morphogenetic proteins in skeletal tissue engineering and regeneration. Nat Biotechnol.

[73] Ogasawara T, Kawaguchi H, Jinno S (2004). Bone morphogenetic protein 2-induced osteoblast differentiation requires Smad-mediated down-regulation of Cdk6. Mol Cell Biol.

[74] Ivanova V, Chenchev I, Zlatev S, Mijiritsky E (2021). Comparison study of the histomorphometric results after socket preservation with PRF and allograft used for socket preservation-randomized controlled trials. Int J Environ Res Public Health.

[75] Famili P, Jose SP (2018). Compare combination allograft material to DFDBA in alveolar ridge preservation. J Dent Health Oral Disord Ther.

[76] Gerritsen AE, Allen PF, Witter DJ, Bronkhorst EM, Creugers NH (2010). Tooth loss and oral health-related quality of life: a systematic review and meta-analysis. Health Qual Life Outcomes.

[77] Kalsi AS, Kalsi JS, Bassi S (2019). Alveolar ridge preservation: why, when and how. Br Dent J.

[78] Rosner B (2015). Fundamentals of Biostatistics. https://old.amu.ac.in/emp/studym/100018285.pdf.

[79] Miron RJ, Fujioka-Kobayashi M, Hernandez M, Kandalam U, Zhang Y, Ghanaati S, Choukroun J (2017). Injectable platelet rich fibrin (i-PRF): opportunities in regenerative dentistry?. Clin Oral Investig.

[80] Gangwani KD, Shetty L, Kulkarni D, Seshagiri R, Chopra R (2018). Piezosurgery versus conventional method alveoloplasty. Ann Maxillofac Surg.

[81] Marini L, Rojas MA, Sahrmann P, Aghazada R, Pilloni A (2018). Early wound healing score: a system to evaluate the early healing of periodontal soft tissue wounds. J Periodontal Implant Sci.

[82] Landry RG, Turnbull RS, Howley T (1988). Effectiveness of benzydamyne HCl in the treatment of periodontal post-surgical patients. Res Clin Forums.

[83] Pippi R (2017). Post-surgical clinical monitoring of soft tissue wound healing in periodontal and implant surgery. Int J Med Sci.

[84] Bansal M, Kumar A, Puri K, Khatri M, Gupta G, Vij H (2016). Clinical and histologic evaluation of platelet-rich fibrin accelerated epithelization of gingival wound. J Cutan Aesthet Surg.

[85] Chenchev IL, Ivanova VV, Neychev DZ, Cholakova RB (2017). Application of platelet-rich fibrin and injectable platelet-rich fibrin in combination of bone substitute material for alveolar ridge augmentation - a case report. Folia Med (Plovdiv).

[86] Forsung E, Cameron JA, Ley D, DiDomenico LA (2020). Current Concepts With Autogenous Bone Grafting. Current Concepts With Autogenous Bone Grafting.

[87] Movahed R, Pinto LP, Morales-Ryan C, Allen WR, Wolford LM (2013). Application of cranial bone grafts for reconstruction of maxillofacial deformities. Proc (Bayl Univ Med Cent).

[88] Schmidt AH (2021). Autologous bone graft: is it still the gold standard?. Injury.

[89] Pandit N, Pandit IK, Malik R, Bali D, Jindal S (2012). Autogenous bone block in the treatment of teeth with hopeless prognosis. Contemp Clin Dent.

[90] Sumer M, Keles GC, Cetinkaya BO, Balli U, Pamuk F, Uckan S (2013). Autogenous cortical bone and bioactive glass grafting for treatment of intraosseous periodontal defects. Eur J Dent.

[91] Borg TD, Mealey BL (2015). Histologic healing following tooth extraction with ridge preservation using mineralized versus combined mineralized-demineralized freeze-dried bone allograft: a randomized controlled clinical trial. J Periodontol.

[92] Nelson AC, Mealey BL (2020). A randomized controlled trial on the impact of healing time on wound healing following ridge preservation using a 70%/30% combination of mineralized and demineralized freeze-dried bone allograft. J Periodontol.

[93] Zellner JW, Allen HT, Kotsakis GA, Mealey BL (2023). Wound healing after ridge preservation: a randomized controlled trial on short-term (4 months) versus long-term (12 months) histologic outcomes. J Periodontol.

[94] Jaiswal Y, Kumar S, Mishra V, Bansal P, Anand KR, Singh S (2017). Efficacy of decalcified freeze-dried bone allograft in the regeneration of small osseous defect: a comparative study. Natl J Maxillofac Surg.

[95] Wei L, Miron RJ, Shi B, Zhang Y (2015). Osteoinductive and osteopromotive variability among different demineralized bone allografts. Clin Implant Dent Relat Res.

[96] Gothi R, Bansal M, Kaushik M, Khattak BP, Sood N, Taneja V (2015). A comparative evaluation of freeze dried bone allograft and decalcified freeze dried bone allograft in the treatment of intrabony defects: a clinical and radiographic study. J Indian Soc Periodontol.

[97] Wu DT, Munguia-Lopez JG, Cho YW, Ma X, Song V, Zhu Z, Tran SD (2021). Polymeric scaffolds for dental, oral, and craniofacial regenerative medicine. Molecules.

[98] Wood RA, Mealey BL (2012). Histologic comparison of healing after tooth extraction with ridge preservation using mineralized versus demineralized freeze-dried bone allograft. J Periodontol.

[99] Clark D, Rajendran Y, Paydar S (2018). Advanced platelet-rich fibrin and freeze-dried bone allograft for ridge preservation: a randomized controlled clinical trial. J Periodontol.

[100] Perez LA, Brooks SL, Wang HL, Eber RM (2005). Comparison of linear tomography and direct ridge mapping for the determination of edentulous ridge dimensions in human cadavers. Oral Surg Oral Med Oral Pathol Oral Radiol Endod.

[101] Lu JW, Shi X, Huang SH, Yan XZ, Hu CJ, Shi MY, Luo LJ (2022). A novel cone-beam CT scanning technique for measuring periodontal soft tissues in the esthetic area. J Oral Sci.

[102] Khazaal Al-Jaboori AS, Hassan NA (2023). Cone beam CT assessment of bone width of upper and lower jaws for dental implant placement: an Iraqi study. Scientifica (Cairo).

[103] Ranganathan M, Balaji M, Krishnaraj R, Narayanan V, Thangavelu A (2017). Assessment of regeneration of bone in the extracted third molar sockets augmented using xenograft (CollaPlug(TN) Zimmer) in comparison with the normal healing on the contralateral side. J Pharm Bioallied Sci.

[104] Wang X, Zhang Y, Choukroun J, Ghanaati S, Miron RJ (2018). Effects of an injectable platelet-rich fibrin on osteoblast behavior and bone tissue formation in comparison to platelet-rich plasma. Platelets.

[105] Thanasrisuebwong P, Surarit R, Bencharit S, Ruangsawasdi N (2019). Influence of fractionation methods on physical and biological properties of injectable platelet-rich fibrin: an exploratory study. Int J Mol Sci.

[106] Kyyak S, Blatt S, Pabst A, Thiem D, Al-Nawas B, Kämmerer PW (2020). Combination of an allogenic and a xenogenic bone substitute material with injectable platelet-rich fibrin - a comparative in vitro study. J Biomater Appl.

[107] Gülşen U, Dereci Ö (2019). Evaluation of new bone formation in sinus floor augmentation with injectable platelet-rich fibrin-soaked collagen plug: a pilot study. Implant Dent.

[108] Cameron CE (1964). Cracked-tooth syndrome. J Am Dent Assoc.

[109] Cameron CE (1976). The cracked tooth syndrome: additional findings. J Am Dent Assoc.

[110] Lee CYS, Prasad H, Lee CCY, Prasad S, Suzuki JB (2020). ﻿Positive effect of injectable platelet rich fibrin (i-PRF) on vital bone formation in graft reconstruction of the mandible: a histologic and histomorphometric study. Int J Dent Res Rev.

[111] Kemmochi M, Sasaki S, Takahashi M, Nishimura T, Aizawa C, Kikuchi J (2018). The use of platelet-rich fibrin with platelet-rich plasma support meniscal repair surgery. J Orthop.

[112] Agrawal AA (2023). Platelet rich fibrin is not a barrier membrane! Or is it?. World J Clin Cases.

[113] Thakkar DJ, Deshpande NC, Dave DH, Narayankar SD (2016). A comparative evaluation of extraction socket preservation with demineralized freeze-dried bone allograft alone and along with platelet-rich fibrin: a clinical and radiographic study. Contemp Clin Dent.

[114] Soni R, Priya A, Yadav H, Mishra N, Kumar L (2019). Bone augmentation with sticky bone and platelet-rich fibrin by ridge-split technique and nasal floor engagement for immediate loading of dental implant after extracting impacted canine. Natl J Maxillofac Surg.

